# Bayesian Analysis of Three Methods for Diagnosis of Cystic Echinococcosis in Sheep

**DOI:** 10.3390/pathogens9100796

**Published:** 2020-09-27

**Authors:** Piero Bonelli, Federica Loi, Maria Giovanna Cancedda, Angela Peruzzu, Elisabetta Antuofermo, Elisabetta Pintore, Toni Piseddu, Giovanni Garippa, Giovanna Masala

**Affiliations:** 1 OIE Reference Laboratory for Echinococcosis, National Reference Center for Echinococcosis (CeNRE), IZS della Sardegna, 07100 Sassari, Italy; angela.peruzzu@izs-sardegna.it (A.P.); toni.piseddu@izs-sardegna.it (T.P.); giovanna.masala@izs-sardegna.it (G.M.); 2 OEVR-Osservatorio Epidemiologico Veterinario Regionale della Sardegna, IZS della Sardegna, 09123 Cagliari, Italy; federica.loi@izs-sardegna.it; 3 Anatomical Pathology, Histopathology, Animal Genetics Laboratory, IZS della Sardegna, 07100 Sassari, Italy; mariagiovanna.cancedda@izs-sardegna.it; 4 Department of Veterinary Medicine, University of Sassari, 07100 Sassari, Italy; eantuofermo@uniss.it (E.A.); elisa.pintore@gmail.com (E.P.); garippa@uniss.it (G.G.); 5 Mediterranean Center for Disease Control (MCDC), University of Sassari, 07100 Sassari, Italy

**Keywords:** cystic echinococcosis, *Echinococcus granulosus*, diagnostic accuracy, Bayesian latent class analysis

## Abstract

Diagnosis of cystic echinococcosis (CE) in sheep is essentially based on necropsy findings. Clinical symptoms can be easily overlooked, while the use of immunological tests is still not recommended for an intra vitam diagnosis. This study assessed the performances of three post-mortem laboratory methods in the diagnosis of ovine CE. In the absence of a single and accurate test as a gold standard, the results of multiple analytical tests can be combined to estimate diagnostic performance based on a Bayesian statistical approach. For this purpose, livers (*n* = 77), and lungs (*n* = 79) were sampled from adult sheep and examined using gross pathology, histopathology and molecular analyses. Data from the three diagnostic methods were analyzed using a Bayesian latent class analysis model to evaluate their diagnostic accuracy in terms of sensitivity (Se), specificity (Sp), positive predictive value (PPV) and negative predictive value (NPV). The gross pathology examination revealed excellent diagnostic capabilities in diagnosing ovine CE with an Se of 99.7 (96.7–99.8), Sp of 97.5 (90.3–99.8), PPV of 97.6 (90.5–100), and NPV of 99.7 (96.5–100). The experimental design used in this work could be implemented as a validation protocol in a quality assurance system.

## 1. Introduction

Cystic echinococcosis (CE) is a globally-distributed zoonosis caused by the larval form of *Echinococcus granulosus sensu lato* (*s.l*.). Due to its severe clinical implications, human CE is considered a public health concern worldwide. The *E. granulosus s.l.* life cycle includes definitive (wild and domestic carnivores) and intermediate hosts (wild and domestic ungulates). Definitive hosts cause environmental contamination with parasite eggs shed in the feces, which are ingested by intermediate hosts and develop into larvae or metacestodes (hydatids). Metacestode encystment within infected organs can cause clinical problems associated with the growth and localization of the hydatid cysts, most frequently occurring in the liver and lungs. Humans are considered aberrant intermediate hosts, acquiring the infection by accidental ingestion of eggs. In countries, where sheep farming is important for the local economy, CE is caused by *E. granulosus sensu stricto* (*s.s*) and maintained primarily by a dog–sheep cycle [1]. An *E. granulosus s.s.* infection in sheep tends to be asymptomatic in many cases [2], such that it is regularly diagnosed post-mortem during veterinary inspection at slaughterhouses. To date, the absence of an alternative and reliable intra vitam diagnostic method able to detect *E. granulosus s.s.* infection has hampered the effectiveness of control and surveillance measures. Although serological assays combined with imaging techniques are commonly used for the diagnosis of human CE [3], their application in sheep presents important issues of sensitivity and specificity, especially due to the absence of standardized antigens and the frequent occurrence of co-infection with other parasites sharing cross-reacting epitopes [4,5]. Ultrasound examination in sheep is reliable for the detection of hydatid cysts in the liver [6], but is not as sensitive for lungs lesions. Thus, ultrasound cannot be considered for practical use since it is time-consuming and requires specialized skills and diagnosis of CE in intermediate hosts and in sheep, it relies essentially on post-mortem examination. Moreover, macroscopical examination of internal organs, mainly the liver and lungs, can be considered a reference test used for the surveillance of control programs [4] and vaccination trials [7], although it can be biased by low sensitivity in younger animals [8,9]. However, early developmental stage and degeneration processes of the hydatid cysts may limit the diagnostic power of gross pathology. In such circumstances, microscopic observation of suspect lesions can be useful to detect *E. granulosus* by evidencing characteristic histopathological patterns of metacestode stage. Besides, several DNA techniques, mainly based on PCR, are also available [4], allowing the identification of *E. granulosus* from cyst material even though molecular approaches are more addressed for studies of genetic variation and molecular epidemiology [10].

The accuracy of diagnostic tests is usually defined against an established gold standard. In several cases, the gold standard test may not be available, of unknown accuracy, too invasive, or imperfect, generating imprecise sensitivity and specificity values [11]. In the absence of a single and accurate gold standard, a combination of multiple analytical methods can be used to estimate the diagnostic performance of a test utilizing a frequentist or Bayesian statistical approach. When the disease status of the individuals is unknown or latent, the traditional class of reference models are the latent class models (LCMs) [12]. Nowadays, the usage of the Bayesian approach in LCMs to evaluate the accuracy of laboratory methods, both for human and veterinary diagnostics, has become increasingly widespread [13,14,15,16,17,18,19].

This study applied Bayesian LCMs to evaluate gross pathology (GP) in comparison with histopathology (H) and molecular analysis (MA, amplification and sequencing of *cox 1* mitochondrial gene) for CE diagnosis in sheep. The diagnostic accuracy of the three laboratory tests were separately determined.

## 2. Results

A total of 156 organs from 79 slaughtered sheep were examined to detect larval forms of *E. granulosus.* Table 1 shows the frequencies of the three tests’ results. Thirty-nine out of 77 livers (50.6%) and 42 out of 79 lungs (53.2%) were found positive by GP. All three tests were found to be concordant in 47.6% (95% IC = 44.3–48.9) of the positive and 47.4% (95% IC = 44.5–49.6) of the negative samples. GP and H failed to detect only one sample found to be positive by MA. Comparing GP and H, the tests were compatible in the 50.6% positivity in livers without significant statistical differences (χ^2^ = 22.15, *p* = 0.08), while a lower positivity rate of 45.6%was detected in the lungs (χ^2^ = 11.87, *p* = 0.0005). In contrast, MA evidenced lower positivity rates than GP for both livers (23.4%, χ^2^ = 50.63, *p* < 0.0001) and lungs (30.4%, χ^2^ = 39.77, *p* < 0.0001). 

The proportion of positive samples detected by the three methods was subdivided based on the different morphological characteristics of hydatid cysts, as reported in Table 2. The three laboratory tests gave perfectly concordant results (100%) in the identification of fertile hydatid cysts. H showed no statistically-significant differences from GP in discriminating the different types of hydatid cysts. MA, however, had a lower ability than GP in detecting non-fertile (52.4%, χ^2^ = 13.12, *p* = 0.0003) transitional (42.8%, χ^2^ = 16.51, *p* < 0.0001) and, especially, inactive cysts (13%, χ^2^ = 34.38, *p* < 0.0001). 

Sensitivity (Se), specificity (Sp), positive predictive value (PPV), and negative predictive value (NPV) for each diagnostic method with 95% credibility intervals estimated by the Bayesian LCMs with the subject random effect are presented in Table 3. It must be noted that H was not performed on negative samples on GP, which means no suspect lesions could be sampled, assuming that H was negative too and setting Sp and PPV equal to 1 in these cases.

**Figure 1:**
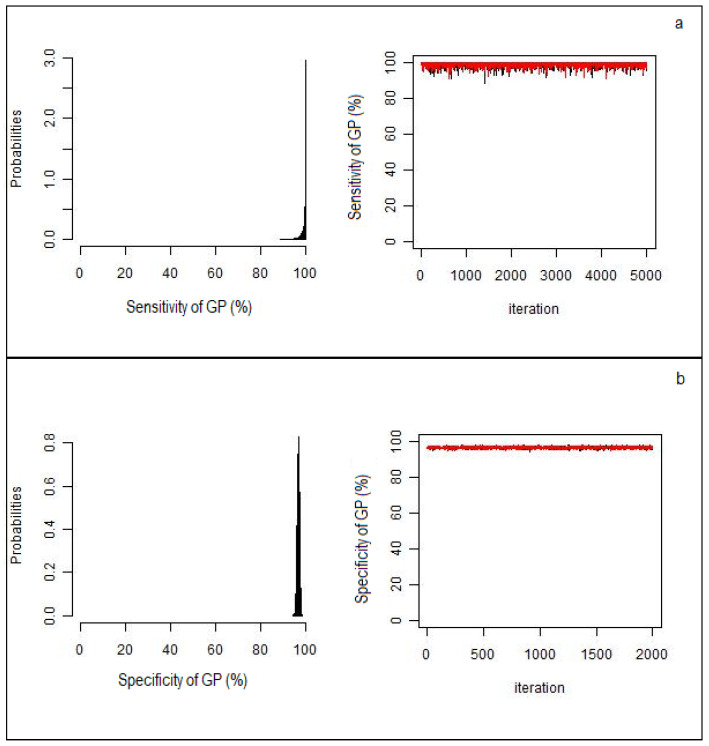
Black histograms describing sensitivity (**a**) and specificity (**b**) of gross pathology (GP), with associated the Markov chain distributions (red and black). All the chains converged confirming that the estimated parameters by the Bayesian model are reliable.

GP had better Se, PPV, and NPV than H or MA. Higher specificity values were found for MA because of a single sample detected positive in MA but negative in GP. All the chains simulated by Bayesian LCMs converged, indicating a reliable results for estimated parameters (Figure 1a,b).

**Figure 2:**
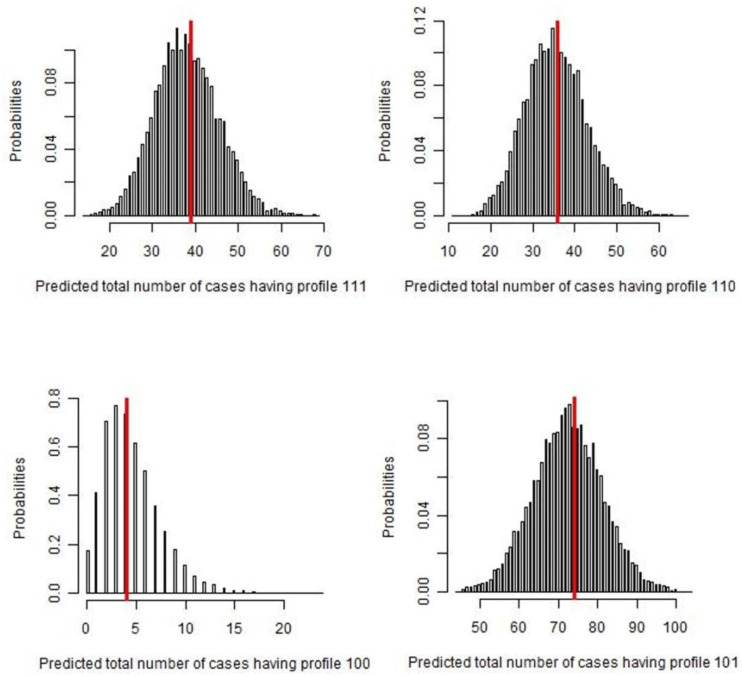
Number of cases predicted by the Bayesian latent-class analysis, for each GP profile (111, 110, 100, 101). The red line represents the observed frequency of each test result profile, while the histograms illustrate the predictive posterior distribution of predicted frequency. In each of the figures, the dataset was replicated 50,000 times and selected only 5000 times (thin sampling equals to 10) to assess the probability of observed frequencies, assuming the model was true.

As expected, considering the parameter estimates obtained, the best agreement between “frequency observed” and “frequency predicted” using Bayesian *p*-values and posterior predictive distribution was shown by the GP-positive profile (111, 110, 101, 100), with a *p*-value near 0.5 (Figure 2).

## 3. Discussion

This study aimed to evaluate the diagnostic performance of three post-mortem laboratory methods (gross pathology, histopathology, and molecular biology) in the diagnosis of CE in sheep. For evaluation, internal organs were collected at slaughterhouses in the endemic Italian region of Sardinia. Livers and lungs were examined by pathological examination to detect the typical lesions of *E. granulosus* infection and subsequently classified according to their morphological characterization. Samples of hydatid cysts were also analyzed by histological and molecular methods. In the absence of a gold standard for CE diagnosis in intermediate hosts, a Bayesian latent class analysis was applied to the laboratory results to separately establish the diagnostic accuracy of the three tests. 

We decided to use this Bayesian approach and to compare methods that are normally used in a complementary way [8,20] because of the great difficulty in discriminate between CE-positive and CE-negative animals with other diagnostic tools. As already stated, serological assays and ultrasound scanning for CE diagnosis cannot confidently identify infected animals [21] that is a basic condition for a diagnostic accuracy study.

Bayesian LCMs can estimate the true sensitivity and specificity of a diagnostic test without the need for a gold standard [22]. Due to the unknown accuracy of the gold standard, the variable of “true” disease status is included in the class model. This variable contains two mutually-exclusive categories, “diseased” and “non-diseased”. The real value of this variable is considered unobserved, or latent, conferring the term of “latent class analysis”. The LCM did not assume that any test is perfect; instead, it considered the true accuracy of each test for diagnosing the true disease status. Even when the model cannot determine the “true” disease status of each subject, the chance of having “true” disease in each subject (prevalence) can be determined [12]. 

The analyses of 156 organs gave different positivity rates based on the diagnostic tests performed. The highest positivity percentages were found by GP in the liver and lungs. In comparison with GP, H found the same number of positive livers but could not detect five positive lungs infected with non-fertile (*n* = 1), transitional (*n* = 1), or inactive cysts (*n* = 3). Considering that histological examinations were not performed in samples that did not contain cysts, the Sp value in such cases was fixed to 1 to carry out the Bayesian LCM. Consequently, the higher PPV value of H than GP cannot be considered relevant for the best performance definition. Sensitivity analysis, which assessed the agreement between “frequency observed” and “frequency predicted” using the Bayesian *p*-value and posterior predictive distribution of each profile, confirmed that GP has a higher ability in positivity detection as reported in Figure 2. Since the red line represents the observed frequency of each test result profile, and the histograms illustrate the predictive posterior distribution of the predicted frequency assuming the model is true, the probability of observed frequencies strictly filled in the predicted ones.

In this study gross pathology was revealed to have excellent diagnostic performance in the diagnosis of ovine CE. Our data showed that the performance of H was hampered by the degenerative process of hydatid cysts, which compromised the correct detection of microscopic patterns indicative of *E. granulosus* infection. Heavy infiltration of inflammatory cells and mediators causes important changes in cyst architecture [23,24], markedly modifying germinal layer structure until its complete disappearance and causing enough fragmentation of the laminated layer such that it is no longer recognizable in some histological sections [25,26].

Likewise, the lower positivity percentages of MA than GP, as we observed especially in inactive cysts (13% vs. 95.6%), may be attributed to the presence of PCR inhibitors (Schrader) in the degenerated lesions or the DNA degradation caused by inflammatory processes. It has been reported that Th1 effector cells recruited to the site of infection have a role in the process of hydatid cyst degeneration [27]. In particular, the production of free radicals induced by IFN-γ [28] are responsible for oxidative damage to many cellular molecules including DNA [29]. Fragmentation of parasite DNA may have affected our PCR-based methodology, preventing proper amplification of the 800 bp target. The design of different primers to shorten the amplicon length, thus reducing the likelihood of DNA polymerase to encounter template disruptions, would have possibly improved the diagnosis of hydatid cysts as reported for other cestode species [30,31].

## 4. Materials and Methods 

### 4.1. Sample Collection

A total of 79 adult Sardinian sheep (aged >3 years) were included in this study. After slaughter at local abattoirs, 156 organs (77 livers and 79 lungs) were collected. Gross pathology examination was performed to detect cystic lesions attributable to *E. granulosus*. Hydatid cysts were excised from each positive organ and divided in two parts for subsequent histological and molecular analyses based on PCR test.

### 4.2. Gross Pathology (GP)

Livers and lungs were macroscopically examined to detect hydatid cysts. Organs were cut at intervals of approximately 3–5 mm for the detection of cysts in the parenchyma. A hydatid cyst was considered a fluid-filled cystic structure consisting of three layers—an inner germinal layer, a middle laminated layer, produced by the parasite, and an outer adventitial layer, generated by the host inflammatory response [8]. For cysts with a cavity and hydatid fluid, the contents were aspirated and observed under a stereomicroscope (4×) to detect protoscoleces. Hydatid cysts were classified as “fertile” or “non-fertile” based on the presence or absence of protoscoleces, respectively. Hydatid cysts with a degenerative or calcified content were classified as “inactive”, while those with intermediate morphological features were classified as “transitional”. If no cystic lesions were found, organs were homogenized in a mixer for molecular analyses. 

### 4.3. Histopathology (H)

Hydatid cysts samples were fixed in 10% neutral buffered formalin and embedded in paraffin following routine laboratory protocols for histopathology. Sections were cut serially from paraffin blocks at 4 µm and stained with hematoxylin and eosin (H&E) and with modified period-acid Schiff (PAS) stains. The presence of an outer PAS-positive acellular laminated layer with an inner cellular nucleated germinal layer was suggestive of metacestodes of the *Echinococcus* spp. [8]. Histology was not performed in samples that did not have hydatid cysts.

### 4.4. Molecular Analysis (MA)

Samples of hydatid cysts and homogenized organs were stored frozen at −80 °C. Total genomic DNA was extracted using the Qiagen DNeasy Blood and Tissue Kit (Quiagen, Hilden, Germany). Molecular identification of *E. granulosus* was performed by amplifying and sequencing a fragment of the mitochondrial gene cytochrome c oxidase subunit 1 (*cox1*) as previously described [32].

### 4.5. Bayesian Latent Class Analysis

Data from laboratory analyses were recorded on a spreadsheet (Excel® Microsoft Corp., Redmond, WA, USA), and positivity rates were calculated for each diagnostic test and each type of cyst (fertile, non-fertile, transitional, or inactive). Differences in positivity rates were statistically tested using the Chi-squared test for independence. Evaluation of the best test for identification of different cyst type was performed by comparing the precision of positive cyst detection by MA and H with respect to GP [33,34]. Data from the three diagnostic methods were analyzed to evaluate their performance for CE diagnosis in terms of sensitivity (Se), specificity (Sp), positive predictive value (PPV) and negative predictive value (NPV), using Bayesian LCMs. For each diagnostic test, the sample was declared “1” if positive and “zero” if negative or not performed. In this manner eight categories were identified—111, 110, 101, 011, 100, 010, 001, and 000. Furthermore, it was assumed that the sensitivity was unknown and, hence, non-informative beta prior distributions of between 0 and 1 were used in the analysis since beta distributions are well suited to describe the uncertainty associated with a binomial probability. Se and PPV of H were fixed to 1 value, considering that samples detected as negative in GP were not analyzed in H. Applying the maximum likelihood estimation method proposed by Hui and Walter [11] and expanded by Walter and Irwig [35], the true accuracy of all three diagnostic tests was estimated. In order to consider the conditional dependence among the positive organs of the same animals and assuming that each subject had a different Se, a continuous random effect normally distributed as *ri* ~ *N*(0,1) was fixed on the final Bayesian LCM [36]. Results are reported as the median estimate and 95% credibility intervals (95% CrI), calculated from the 0.5 and 0.025–0.975 percentiles, respectively, of the posterior distribution. To check the goodness of fit of the model, observed and expected frequency values of each combined category were compared. The Bayesian *p*-values were calculated as the probability that replicate data (predicted frequency) from the Bayesian model were more extreme than the observed data. A Bayesian *p*-value close to 0 or 1 indicates that the observed result would be unlikely in data replication if the mode were true. This means that for Bayesian *p*-values close to or exactly 0.5, the Bayesian model describes the observed data very well. All statistical analyses were performed using R-software, version 3.6.4 (R Development Core Team, Vienna, Austria). The Markov chain Monte Carlo (MCMC) analysis was run using JAGS version 3.1.0 through the R package “*rjags*” [37] with 50,000 iterations and an initial burn-in of 10,000 iterations. The MCMC convergence was assessed by visual inspection and analyzing the difference between multiple Markov chains, as suggested by the Brooks–Gelman–Rubin statistic. The convergence was assessed by comparing the estimated between-chain and within-chain variances for each model parameter. Large differences between these variances indicated non-convergence [38].

## 5. Conclusions

Despite its application in surveillance and control programs, gross pathologic examination had never been proven to be a gold standard for CE diagnosis in intermediate hosts. In this work gross pathology was revealed to be a screening test with excellent diagnostic capabilities in the detecting of hydatid cysts in ovine internal organs. Degeneration processes occurring naturally in cystic lesions can reduce the diagnostic accuracy of histopathologic and molecular testing, Nonetheless, histopathology and molecular analyses are important complementary tools to support diagnosis of ovine CE. DNA techniques based on PCR amplification of long fragments are of better use in molecular characterization studies. The experimental design proposed may be applicable for the development of validation protocols for quality assurance systems.

## Figures and Tables

**Table 1:** Frequencies of results obtained by the three post-mortem laboratory methods for cystic echinococcosis (CE) diagnosis.

Diagnostic Methods			Frequencies (Number of Samples)
GP	H	MA	
Positive	Positive	Positive	39
Positive	Negative	Positive	2
Positive	Positive	Negative	36
Negative	Positive	Positive	0
Positive	Negative	Negative	4
Negative	Negative	Positive	1 *
Negative	Positive	Negative	0 *
Negative	Negative	Negative	74 *

GP, gross pathology; H, histopathology; MA, molecular analysis. * Histopathology (H) was assumed to be negative since it was not performed on samples negative on gross pathology.

**Table 2:** The proportion (percentage, n/total) of CE-positive samples sorted by the different types of hydatid cysts (fertile, non-fertile, transitional, or inactive) detected by three diagnostic tests.

Diagnostic Methods	Hydatid Cysts			
	Fertile	Non Fertile	Transitional	Inactive
GP	100 (16/16)	100 (21/21)	100 (22/22)	95.6 (22/23)
H	100 (16/16)	95.2 (20/21)	95.2 (21/22)	82.6 (19/23)
MA	100 (16/16)	52.4 * (11/21)	42.8 * (10/22)	13 * (3/23)

GP, gross pathology; H, histopathology; MA, molecular analysis. * indicates statistically significant difference (*p* ≤ 0.0003) with the corresponding value obtained by GP.

**Table 3:** Diagnostic accuracy measures with corresponding 95% credibility intervals based on the Bayesian latent class model (LCM) with the random effect for the three tests applied.

Diagnostic Accuracy Measures	Diagnostic Methods		
	GP	H	MA
Se	99.7 (96.7–99.8)	94.7 (87.3–99.2)	51.8 (40.8–62.8)
Sp	97.5 (90.3–99.8)	1 ^§^	98.4 (93.8–99.9)
PPV	97.6 (90.5–100)	1 ^§^	97.2 (89.1–99.8)
NPV	99.7 (96.5–100)	94.8 (87.2–99.3)	66.6 (56.9–74.7)

GP, gross pathology; H, histopathology; MA, molecular analysis; Se, sensitivity; Sp, specificity; PPV, positive predictive value; NPV, negative predictive value. ^§^ Specificity and positive predictive value of H were fixed to 1.
